# Ethnomedicinal Evaluation of Medicinal Plants Used against Gastrointestinal Complaints

**DOI:** 10.1155/2015/892947

**Published:** 2015-05-31

**Authors:** Akash Tariq, Sakina Mussarat, Muhammad Adnan, E. F. Abd_Allah, Abeer Hashem, Abdulaziz Abdullah Alqarawi, Riaz Ullah

**Affiliations:** ^1^Department of Botany, Kohat University of Science and Technology, Kohat 26000, Pakistan; ^2^Plant Production Department, College of Food and Agricultural Sciences, King Saud University, Riyadh 11451, Saudi Arabia; ^3^Botany and Microbiology Department, College of Science, King Saud University, Riyadh 11451, Saudi Arabia; ^4^Mycology and Plant Disease Survey Department, Plant Pathology Research Institute, Agriculture Research Center, Giza 2109, Egypt; ^5^Department of Chemistry, Government College Ara Khel, Frontier Region Kohat 26000, Pakistan

## Abstract

Aim of the present study was to document ethnomedicinal plants used against gastrointestinal complaints in five selected remote regions of Pakistan and to select potential medicinal plants for further *in vitro* and *in vivo* investigation. Data on ethnomedicinal plants and ethnographic profile of respondents was documented using semistructured questionnaires. The present study revealed utilization of 52 medicinal plants for the treatment of different gastrointestinal infections in studied regions. Apiaceae was the most dominant family reported to be used for the treatment of these infections (4 plants). Among all the plant parts fruit (24%), whole plants and leaves (23% each) were the most preferred plant parts used by the healers. Dosage of recipe was found to be related with the age of the patient. Highest degree of informant consensus was reported for vomiting, nausea (0.92 each), abdominal pain (0.9), and diarrhea (0.89). *Withania coagulans* scored highest FL value (86%) followed by *Mentha longifolia* and *Melia azadirachta* ranked second with FL value (75% each). Young generation was found to possess little traditional knowledge about utilizing plant recipes against these infections. Plants with high Fic and FL values should be subjected for further phytochemical and pharmacological investigation for scientific validation.

## 1. Introduction

Plants are an important resource of conventional medicines used against different ailments. Rural people who have century's old traditional knowledge transferred from generation to generation still rely on plant resources for variety of purposes such as food, fodder, and medicines. Rural area people heavily depend on natural resources due to lack of modern medical facilities [1]. More than 80% of population in Pakistan is dependent on traditional medicines for health practices [2], but now it is restricted to rural areas [3], due to divergence of people toward modern health facilities in urban areas and also due to changing life styles with the passage of time. This centuries old traditional knowledge is facing severe threat due to modernization in rural societies. Total 6000 plant species have been documented in Pakistan among which only 600 plants have been reported in ethnomedicinal studies [4]. It is therefore imperative to increase ethnomedicinal studies in order to preserve this precious knowledge before its extinction [5].

Gastrointestinal disorders are common in Southwest of Khyber Pakhtunkhwa because these areas lack hygienic condition and malnutrition as well as having insufficient availability of pure water. Approximately 103 million people in Pakistan with an annual growth rate of 3% [6] and population density is recorded 151.8 per square kilometer [7]. Major health risks in Pakistan are extensive communicable diseases, insufficient sewage systems, and lack of pure drinking water [8, 9]. The high infant morbidity and mortality rates reflect inadequate nutrition and exposure to polluted water [6]. Most of the infectious diseases are caused by microorganism such as* Vibrio cholerae*,* Escherichia coli*,* Shigella* spp.*, Salmonella* spp.*, Aeromonas *spp.*, Pseudomonas* spp.,* Campylobacter* spp.,* Klebsiella* spp., and* Staphylococcus aureus* [10]. However, antibiotic resistance is a major clinical problem in treating infections caused by these microorganisms. Synthetic drugs such as proton pump inhibitors, H2 receptors, cytoprotectants, demulcents, anticholinergics, antacids, and prostaglandin analogues are used for the treatment of gastro problems but these drugs produce several side effects. Herbal remedies are considered as better alternatives for the treatment. For example, proton pump inhibitors (omeprazole, lansoprazole) may cause nausea, abdominal pain, constipation, and diarrhea and H2 receptor antagonists (cimetidine) may cause gynaecomastia and loss of libido. Due to the occurrence of many side effects by use of synthetic drugs for many diseases, medicinal plants are considered as the main source of new drugs as they have less or no side effects. Herbal medicines are considered as safe with lesser adverse effects; economical, effective, relatively less toxic, and extensive research is carried out in search for potent drugs of plant origin [11]. In Pakistan and other countries a variety of medicinal plants are used against gastrointestinal complaints such as diarrhea, dysentery, and cholera. However, many of them have not been screened for their phytochemistry and pharmacological action against microbes, which could support their use in traditional medicines.

The present study was the first effort to target gastrointestinal infections and their traditional recipes in five remote southern regions of Khyber Pakhtunkhwa province of Pakistan where these infections were found more common. The main purpose of the study was to conserve the ethnomedicinal knowledge and to select candidate medicinal plants for further phytochemical and pharmacological investigation. The available literature shows that such studies can constitute the starting point for the development of new drugs [12, 13]. Our efforts are towards not only providing nutrition and health care to the people, but also recovering record and diffuse local botanical knowledge and traditional wisdom.

## 2. Material and Methods

### 2.1. Study Area

**Figure 1:**
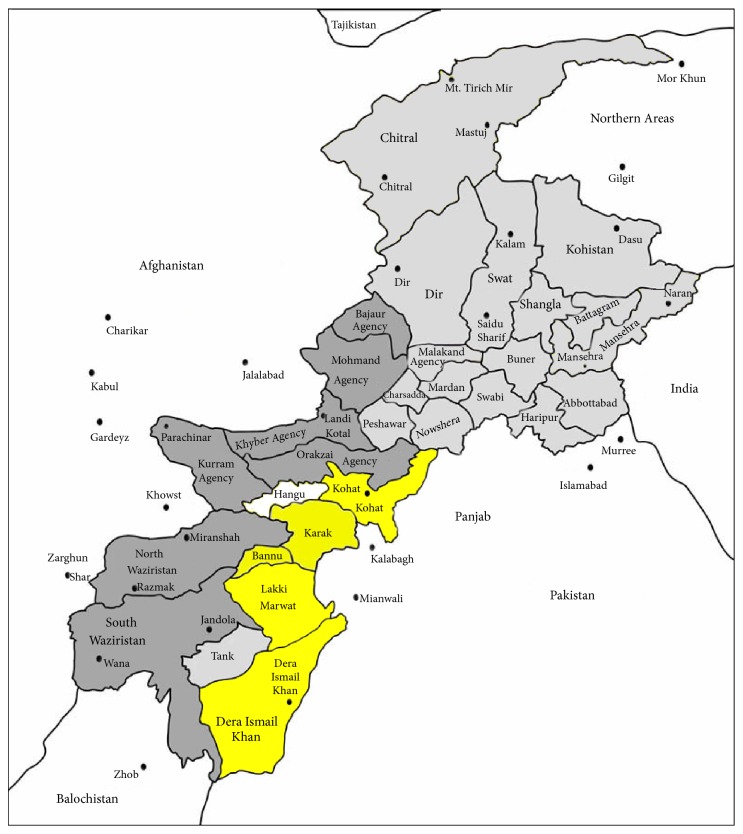


The present study was conducted in five major remote areas (Dera Ismail Khan, Bannu, Lakki Marwat, and Karak and Kohat) of Khyber Pakhtunkhwa province of Pakistan (Figure 1). D. I. Khan is an area of 7326 square kilometers and is situated between 31°15′ and 32°32′N latitude and between 70°11′ and 71°20′E longitude. Most of the area of D. I. Khan is flat dry alluvial plains supporting mostly xerophytic vegetation. Dominant plant species are* Acacia modesta*,* Acacia nilotica*,* Calotropis procera*,* Morus alba,* and* Eucalyptus camaldulensis*. Most of the population of the area is rural with low literacy rate and they also lack modern health facilities; hence, they are more dependent upon natural resources especially plants for their healthcare and to compensate their low income as well [14]. Bannu consists of a total area of 877 square kilometers with a population of 19,593. It lies within the Karakoram mountain range between 32°43 to 33°06N latitude and 73°20 to 70°07E longitude. The total cultivated area is about 33,000 acres, with wheat, maize, and sugarcane being the main cultivated crops. About 25% of the inhabitants of the area as well as Afghan refugees are engaged in the collection and marketing of medicinal plants. The area consists of alluvial plain with an annual rainfall of 111.36 mm. The dominant plant species are* Acacia modesta*,* Acacia nilotica*,* Calotropis procera*,* Dodonaea viscosa,* and* Withania somnifera* [15]. Karak region is situated in the south of KPK with total area of 600 square kilometers and lies between 70-40° to 71-30°N latitude and 32-48° to 33-23°E longitude. The study area is divided into mountainous area, small hills, and plains having most of clayey or sandy soil. Wheat, corn, and gram are the common cultivated crops. Various plant species dominated the study area such as* Acacia modesta, Acacia nilotica, Adhatoda vasica, Aerva javanica, Dodonaea viscosa, Eucalyptus lanceolatus, Fagonia cretica, Rhazya stricta, Saccharum arundinaceum, Withania coagulans,* and* Withania somnifera* [16]. Kohat is located at 33°35′13N, 71°26′29E, with an altitude of 489 m above sea level. The dominant vegetation of the study area is* Zizyphus* species,* Acacia* species, and other xerophytes plants. The area is rural in nature and inhabitants are very much dependent on plants for agricultural, economic, and food purposes. Locals of the region use a variety of medicinal plants for the treatment of various ailments due to expensive modern drugs [17]. Lakki Marwat is situated between 32°161N latitude and 70°191E longitude at altitude of 200–1000 m above sea level. This district covers an area of 3164 km^2^ with a cultivated area of approximately 116,900 ha. The indigenous people of the district are Marwat tribes, but a small proportion of other tribes also settled here. Transport and minerals are the main sources of economy in the urban area, and agriculture is the primary livelihood of the rural population. The major ethnic group in this district speaks Pashto (99.3%), which is spoken in a specific dialect [18]. The remaining population speaks Punjabi (0.7%) in Hindko dialect.

### 2.2. Sampling and Data Collection

Data collection was carried out from 2013 to 2014. Prior to data collection local administrative officers of the regions were visited and it was explained them the main idea of the study in order to get their permission. According to the information provided by the local administrative officers 350 respondents were selected in five studied region with 70 informants in each region. The selection criterion of informants was mainly based on their rich indigenous knowledge and long term experience of utilization of plants as well as their living period of time in the study area. Selected respondents of the regions were aged between 30 and 79 years. Verbal consent, including consent for publication was received from all the informants before the interviews began. The informants are aware that the information they have provided will be published and that data will be used only for scientific purposes. Data was collected in local language (Saraiki, Hindko, and Pashto) and converted into English. Semistructured questionnaires were designed to collect ethnomedicinal knowledge of medicinal plants used against gastrointestinal infections.

### 2.3. Plants Collection and Identification

Plants were collected with the help of respondents from wild and cultivated areas. Collected voucher specimens were taken to the Herbarium of Kohat University of Science and Technology (KUST), Kohat, Pakistan. Specimen identification and confirmation were undertaken by using Flora of Pakistan and taxonomic experts. Specimens with their label were stored at the Herbarium of KUST, Kohat, Pakistan.

### 2.4. Data Organization

The collected data on ethno medicinal plants and ethnography of the respondents was organized using Microsoft Excel 2007 and summarized using graphical statistical methods such as percentages. The habit of the plants was categorized into 3 classes (herbs, shrubs, and trees). Life form of medicinal plants was classified into annual, biennial, and perennial. Plant parts use was categorized into leaves, roots, stem, whole plant, seeds, fruit, and flower. Stomach disorders were divided into six major categories that is, diarrhea, dysentery, abdominal pain, intestinal worms, constipation, and nausea and vomiting.

### 2.5. Data Quality Assurance

Each respondent was approached at least three times during data collection for 5 the legitimacy of information they provided. Information was considered irrelevant and rejected in case of any divergence from the original information of the respondent. Valid data was only subjected to further analysis process. Further data quality was ensured through proper training of data collectors, point out missing information, duplication of material, and careful analysis.

### 2.6. Data Analysis

#### 2.6.1. Informant Consensus Factor (Fic)

Fic was used for the general uses of plants in different study areas and to indicate plants of particular interests. Informants' consensus is the most preferred method to highlight widely used plants for a particular ailment and help in the selection of plants for pharmacological and phytochemical studies [19]. Before using this method, diseases were classified into categories, as high Fic plants are likely to be more pharmacologically active in comparison with low Fic value plants [20]. Fic values lie between “0.00 and 1.00.” When single plant or few plants are used by large number of informants to cure a specific disorder score high Fic values, low Fic values give an indication that informants do not agree over which plant to use [21]. The Fic can be calculated using the formula as follows:

(1) $$\begin{array}{c} Fic=\frac{nur-nt}{nur-1}, \end{array}$$

where Fic = informants consensus factor, nur = number of use citation in each category, and nt = number of species used.

#### 2.6.2. Fidelity Level (FL)

Fidelity level (FL) is useful for recognizing the most favored plants used for curing a special ailment by the respondents [22]. FL values of highly preferred plants are greater than values of less preferred plants. FL values are always calculated in terms of informant's percentage claiming the use of a definite plant species for the same ailment. The FL values indicate the importance of certain plant species for particular purpose. All of the reported ailments grouped into major classes for the calculation of FL values. FL values were estimated by using the formula

(2) $$\begin{array}{c} FL=\frac{Ip}{Iu}\times 100, \end{array}$$

where Ip represents the number of respondents who reported the medicinal plants utilization for a particular ailment and Iu is the total number of respondents who mentioned the same plant for any ailment. It is assumed that those medicinal plants which are used frequently by most respondents for the same category are more likely to be biologically active plants [23].

## 3. Results

In studied regions 52 plants belonging to 36 families (Table 1) were found to be used against gastrointestinal ailments. D. I. Khan region contained high number of medicinal plants (19) for gastrointestinal ailments followed by Bannu (18), Kohat (15), Lakki Marwat (13), and Karak (8). Most dominant family used against gastrointestinal complaints was Apiaceae (4 plants) followed by Cactaceae, Euphorbiaceae, Malvaceae, and Rosaceae (3 plants each) and Meliaceae, Brassicaceae, and Solanaceae (2 plants each). Fruit was the most preferred plant part (24%) used in herbal formulation followed by whole plant (23%) and leaves (19%) (Table 2). Growth form indicated that herbs (50%) were dominating followed by trees (31%) and shrubs (19%). Gastrointestinal disorders were divided into 6 major categories, namely, diarrhea, dysentery, abdominal pain, intestinal worms, constipation, nausea, and vomiting. Mostly wild plants (60%) were used in studied regions as compared to cultivated plants (40%). Most of the herbal recipes were taken orally in decoction or powder form with water, salt, and sugar. It was observed that dosage of the recipe depends upon the age of the patient. Recovery time of these formulations was reported in range of 1–3 days (Table 1).

Fic values for gastrointestinal problems were estimated in range of 0.86 to 0.92. Highest degree of informant consensus was recorded for vomiting and nausea (0.92), abdominal pain (0.9), and diarrhea (0.89). The highest plant use citation was recorded for abdominal pain (191) followed by diarrhea (151) (Table 3). The present study revealed seven potential medicinal plants scoring high FL values.* Withania coagulans* ranked first score highest FL value (86%) followed by* Mentha longifolia and Melia azadirachta* (75% each) ranked second,* Citrullus colocynthis* ranked third with FL value (72%), and* Rosa indica* ranked fourth with (66%) FL value (Table 4).

Demographic data showed highest degree of male informants (57%) followed by female (43%). Majority of the respondents (100) interviewed were 40–49 years age range followed by 50–59 (90) years old (Table 5). Large proportion of informants were illiterate (44%) and the major occupations of male respondents in studied regions were farming, shopkeeping, and females were mostly house wives.

## 4. Discussion

### 4.1. Medicinal Plants Diversity

Natural sources remained an effective method of treatments since the earth was made. The present study revealed high number of plant used against gastrointestinal infection in selected regions of Pakistan that might be due to the highest prevalence of these infections in studied regions. Various ethnomedicinal studies conducted in studied regions proved the presence of great diversity of medicinal plants and occurrence of gastrointestinal infections [14, 15, 24]. Present finding is similar with the studies conducted elsewhere in other countries [25, 26].

### 4.2. Plant Families and Growth Form

Local healers mostly used plants that belong to family Apiaceae because this family has a unique place in homemade remedies and most of its plants are traditionally being used against various gastrointestinal infections not only in Pakistan but throughout the world [27] that might be due to presence of potential phytochemical. Rosaceae, Malvaceae, and Euphorbiaceae are also used by the healers after Apiaceae and similar results have also been reported by [28]. Present findings are contradictory with another ethnomedicinal study conducted somewhere else in which Asteraceae was found to be most frequently used plant family against digestive troubles [29]. These differences among the use of different families among different cultures might be associated with the dominant native vegetation of different areas or might be due to different traditional beliefs. Herb was the dominant growth form used by traditional healers for ethnomedicinal preparations and literature review also proved that herbs are the most widely used growth form worldwide [30, 31].

### 4.3. Traditional Recipes Formulation

Traditional healers used all plant parts in remedy preparation but fruit and whole plant use was most frequent. Possible reason behind these results might be that whole plant and ripened fruit contain high concentration of secondary metabolites. Present results are in line with study conducted in another country in which fruit is most commonly used part against gastrointestinal problems [28] while contradictory with other studies in which leaves are commonly used against digestive problems [31, 32]. Often different parts of a single plant may be concocted and used for a particular type of ailment. For example, the leaves and fruit of* Withania coagulans* are used to treat gastric problems and abdominal pain.* Withania coagulans* also ranked first in present study score highest FL value (86%). This species could be further subjected to phytochemical and pharmacological investigation for proving its efficacy. Decoction was found largest mode of recipe preparation in studied regions and different studies also reported that decoction and infusion are the methods mostly used for the preparation of the folk medicine [15]. Traditional healers of studied regions used variety of vehicles such as sugar, salt, honey, and oil to in herbal formulation and intake of these preparations in order to minimize the bitter taste of plants and avoid vomiting.

### 4.4. Gastrointestinal Infections Treated in the Selected Regions

Most treated diseases in the studied regions are abdominal pain, vomiting, nausea, and diarrhea because majority of people in the studied regions have little or no access to clean drinking water, which may have increased the occurrence of waterborne diseases [33]. Gastrointestinal problems are not only common in the studied areas but are a common issue for the whole country. Moreover, such diseases can result in higher mortality rates if not treated properly on time [34]. Fic results have also shown high degree of consensus against gastrointestinal infections. Plants having high informant consensus against specific ailments are more likely to be biologically active as compared to plants having less Fic values [35].

### 4.5. Diarrhea

Morbidity and mortality due to diarrhea continues to be a major problem in many developing countries, including Pakistan especially amongst children. Worldwide 78% of children deaths due to diarrhea occur in African and South-East Asian regions [36]. Several pathogens are involved in causing diarrhea such as* Escherichia coli, Vibrio cholerae*,* Aeromonas* spp.*, Shigella* spp.*, Salmonella* spp.*, Pseudomonas* spp.,* Klebsiella* spp.,* Campylobacter* spp., and* Staphylococcus aureus* are most common. Present study explored 18 medicinal plants species being used traditionally for diarrhea in studied regions. Bark decoction of* Albizia lebbeck* is traditionally used against diarrhea and literature review showed that its extracts had shown moderate activity against* Vibrio cholerae*,* Aeromonas hydrophila,* and* Bacillus subtilis*. Ethanolic extract of the stem bark of* Albizia lebbeck* on castor oil-induced diarrhea has significant activity (*p* < 0.05) observed at a dose level of 500 mg/kg [37]. Phytochemical studies revealed that the crude methanol extracts contained phenolics and flavonoids and these compounds have previously been reported to possess antimicrobial activities [10]. Traditionally seeds of* Carum copticum* are used for the treatment of diarrhea in studied region.* In vivo* activity of* Carum copticum* at a dose of 100 mg/kg inhibits the gastrointestinal fluid by 39.90–50.70% [38]. Fresh tea made by boiling leaves of* Mentha longifolia* is considered effective in curing diarrhea. FL results ranked* Mentha longifolia* on second position due to its high citation reports that might be due to its higher efficacy and traditional belief of locals. In a castor oil-induced diarrheal model, the crude extract of* Mentha longifolia* at doses of 100–1000 mg/kg provided 31–80% protection. These data indicate that the antidiarrheal and spasmolytic effects of the crude extract of* Mentha longifolia* are mediated through the presence of CCB-like constituent(s), concentrated in the petroleum spirit fraction and this study provides indirect evidence for its medicinal use in diarrhoea and spasm [39]. Outer covering of* Punica granatum* fruit is dried and crushed and powder is taken with water for diarrhea in studied regions. Methanolic extract of the fruit was tested in castor-oil and magnesium-sulfate induced diarrhea in mice at the doses of 200 and 400 mg/kg body weight revealed significant antidiarrheal activity (*p* < 0.001) and inhibited 31.25% defecation at the dose of 200 mg/kg and 53.75% at the dose of 400 mg/kg in castor oil-induced diarrhea while in magnesium sulfate-induced diarrhea the inhibition of defecation was 45.71 and 57.14% at the doses of 200 and 400 mg/kg, respectively. The antidiarrheal effect of the extract was concentration dependent in both castor oil-induced diarrhea and magnesium-sulfate induced diarrhea. Phytochemical screening of the extract revealed the presence of flavonoids and alkaloids that may play key role in its antidiarrheal activity [40]. Pharmacological evidences of documented antidiarrheal plants suggest the reliability of traditional medicines and also the strong traditional knowledge of the healers.

### 4.6. Dysentery

Dysentery is a medical condition that most commonly occurs in areas where living conditions are crowded and hygiene is poor. Protozoans such as* Entamoeba histolytica* and bacteria (Shigella,* Escherichia coli*) are mainly involved in causing dysentery [41]. Dysentery is caused due to ameobiasis (gastrointestinal infection) by* Entamoeba histolytica* and it affects intestines of about 25% of the world population and considered second infectious diseases next to malaria [42, 43]. Antibiotic metronidazole is used against ameobiasis but reported to produce several mutagenic effects and* in vivo* activities showed that it is carcinogenic to mice and produce several side effects such as nausea, vomiting, dry mouth, metallic taste, abdominal pain, headache, constipation, and diarrhea [44]. Several studies have also reported the resistance action of microbes against metronidazole [45]. Worldwide variety of medicinal plants have shown marked inhibitory effects against different protozoans such as* Entamoeba histolytica* and* Giardia lamblia* with fewer side effects in comparison with allopathic drugs [44, 46]. Present study documented total eight medicinal plants traditionally being used in treatment of dysentery. Reported plants have not yet been tested* in vitro* and* in vivo* against* Entamoeba histolytica* infection. These findings suggest future studies on phytochemical and pharmacological aspects of these plants against protozoan infections. Traditionally fruit of* Phyllanthus emblica* is effective in treatment of diarrhea and dysentery. Methanolic extract of* Phyllanthus emblica* fruit tested* in vitro* against* Shigella dysenteriae* and* Escherichia coli* had shown 24 mm inhibition at 100 mg/mL concentration. Phytochemical analysis of fruit revealed the presence of glycosides, saponins, flavonoids, phenols, proteins, and carbohydrates [47]. Decoction of the bark* Acacia nilotica* is used in diarrhea and pods are grinded to make powder mixed with sugar or honey to treat dysentery. Mashram [48] has observed the antimicrobial activity of* Acacia nilotica*, against* Staphylococcus aureus* and* Escherichia coli*. The leaf and bark extracts showed zone of inhibition between 16 and 15.5 mm, respectively, and found most active against* Escherichia coli*. Phytochemical investigation of* Acacia nilotica* showed presence of different secondary metabolites such as leaf contains apigenin, 6-8-bisD-glucoside, rutin, digestive protein crude protein, and tannins, while bark contains tannin, terpenoids, saponins and glycosides, phlobatannin, gallic acid, protocatechuic acid pyrocatechol, (+)-catechin, (−) epigallocatechin-5,7-digallate, and so forth [49, 50]. There are very few studies present on* in vivo* antiprotozoan and antibacterial activities of documented medicinal plants used against dysentery in studied region [51].

### 4.7. Constipation

Constipation is a medical condition caused by various factors such as excessive intake of antibiotics, less fiber and water intake, and lack of exercise.* Clostridium* is considered the common causative agent of constipation in many cases while there is no clear evidence about the involvement of other microbes [52]. Study reported that 7 plants are used for constipation. Traditionally fruit of* Citrullus colocynthis* is cut and boiled in water and sugar is added to make* Murabba* used for constipation and abdominal diseases in all studied regions. Ripened fruit* Opuntia dillenii* is boiled in water with addition of some sugar and taken orally for the treatment of constipation. However, literature is almost unavailable on the* in vitro* and* in vivo* screening of documented medicinal plants against* Clostridium* species. Studies should be carried out on these medicinal plants that could lead toward some interesting findings and developments.

### 4.8. Abdominal Pain

Abdominal pain is a condition caused by a variety of causes such as constipation, indigestion, gastric problem, appendicitis, menstrual cramps, and stomach ulcer. However, traditional people of studied regions use about twenty medicinal plants for the treatment of abdominal pain condition. Powder of mixture of* Foeniculum vulgare*,* Coriandrum sativum,* and* Anethum sowa* fruit with sugar is considered very effective in reducing abdominal pain and other stomach related complaints. The use of mixtures of plants has recently been recognized to increase the efficacy of herbal medicine due to the presence of variety of secondary metabolites [53].* Withania coagulans* fruits are crushed and mixed with salt and taken orally with water for gastric and abdominal pain troubles. Study conducted by [54] indicated the presence of alkaloids, carbohydrates, proteins, steroids and sterols, anthraquinones, flavonoids, tannins, and saponins. Worldwide ethnomedicinal studies have always provided the basis for the development of new novel drugs which indicates the reliability and efficacy of traditional medicines.

### 4.9. Intestinal Worms

Majority of intestinal worms are obligate parasite in humans and grouped under two major phyla, that is, Platyhelminthes (flatworms) and Nematoda (roundworms) and they have developed various adaptive structures to survive in their hosts [55]. In studied regions very few plants were found to be used traditionally against these human intestinal parasites. Leaves and fruit decoction of* Melia azadirachta* and seeds of* Datura strumarium* is used to remove intestinal worms. Pharmacologically these plants have also been proved for their* in vitro* anthelmintic activities [56, 57]. There are many other plants which are being traditionally used against intestinal worms in other countries and their pharmacological activities have also been proved. As an example a study conducted on ten medicinal plants extracts and their fractions by Ukwubile [55] have shown significant anthelmintic activities among which aqueous extracts showed more prominent activity with increasing concentration, comparable to the standard anthelmintic drug, albendazole. Issue of parasites resistance against commonly using antibiotics is on rise nowadays therefore it is urgent need of developing naturally occurring anthelmintics [58].

### 4.10. Nausea and Vomiting

These are symptoms of other medical conditions such as stomach infection, intestinal blockage, food poisoning, overeating, and appendicitis. In total eight medicinal plants are being used in studied regions against these symptoms. Fic results also showed high consensus which indicates the strong agreement of traditional healers on using specific plants against these symptoms. These plants should be subjected for further analysis to prove their efficacy against common conditions related with these symptoms.

## 5. Conclusions

Gastrointestinal infections are causing great health loss in the remote regions of Pakistan due to their common occurrence. Due to low income status and lack of modern health facilities people of the region are using medicinal plants for these infection. They have centuries old traditional knowledge to prepare different types of effective plant remedies against gastrointestinal disorders. Locals of the region are heavily dependent on these medicinal plants therefore causing serious threats to the abundance of these plants. There is a dire need to protect these medicinal plants before their extinction. Proper training should be given to traditional healers for sustainable collection and utilization of this valuable flora. Focus should be given to current environmental factors causing gastrointestinal problems in the region. Plants with high Fic and FL values should be subjected to further* in vitro* and* in vivo* screening that could lead toward the development of some novel drugs with fewer side effects. Young generation should be mobilized toward learning these practices before the extinction of this knowledge as ethnomedicinal knowledge provide a baseline information to chemists, pharmacists, and pharmacologists for drug developments.

## Figures and Tables

**Table 1:** Medicinal uses of plants for stomach troubles.

Plant and family name	Local name	Habit	Life-span	Plant occurrence status	Part used	Uses and recipes	Dosage and recovery		Place
							Children	Adults	
*Abutilon indicum *(L.) Sweet Malvaceae	Koso beta	S	A	W	Wh. pl	Decoction or powder is used against constipation	1 tea spoon after meal	2–4 tea spoons after meal	Lakki Marwat

*Acacia modesta* Wall. Mimosaceae	Phulahi	T	P	W	Ba	Decoction is made by boiling bark in water or powder of bark is made by grinding the bark for treatment of gas trouble and abdominal diseases	2 spoons after 4 hrs for 1 day	3-4 spoons after 2 hrs for 2 days or depends on severity	D. I. Khan

*Acacia nilotica* (L.) Delile Mimosaceae	Kikar	T	P	W	Po, Ba	Decoction of the bark is used in diarrhea and pods are grinded to make powder mixed with sugar or honey to treat dysentery	Small amount of powder is given for 1-2 days	500 mg given twice a day for 3 days	Kohat

*Achyranthes aspera *L. Amaranthaceae	Ghoshkai	H	P	W	Wh. pl	Its decoction is used in stomach disorder. The juice of the plant is used in abdominal pain, dysentery, and in bowel complaints	Depends on disease severity	Depends on disease severity	Bannu

*Albizia lebbeck *(L.) Benth. Mimosaceae	Sirin	T	P	C	Ba	Bark decoction is used to treat diarrhea	1-2 spoon 3 times a day	1-2 spoon after 3 hrs for 1 day	Bannu

*Allium cepa* L. Liliaceae	Piaz	H	A	C	Bu	Equal amounts of extract of onion bulb and mint are mixed and given for cholera	Half tea spoon of this mixture is taken per hour for a period as needed	One tea spoon of this mixture is taken per hour for a period as needed	D. I. Khan

*Argyrolobium roseum* (Cambess.) Jaub. & Spach Nyctaginaceae	Mukhan Butti.	S	B	W	Wh. pl	Diarrhea			Bannu

*Artemisia absinthium* L. Asteraceae	Afsanteen	H	B	W	L	Leaves are boiled to expel intestinal worms, indigestion, diarrhea, and vomiting	As needed	As needed	Kohat

*Asparagus adscendens *Roxb. Asparagaceae	Safid muesli	H	A	W	R	Ground root is effective for carminative	As needed	As needed	Karak

*Azadirachta indica* A. Juss Meliaceae	Neem	T	P	C	L	Decoction of leaves is taken for digestive and gastric problems	2 tea spoons for one day	2 tea spoons for one day	D. I. Khan, Lakki Marwat

*Bistorta amplexicaulis *(D. Don) Greene Polygonaceae	Masloon, Anjabar	H	P	W	Wh. pl	Constipation			Bannu, Kohat

*Boerhaavia diffusa *Brandegee Nyctaginaceae	Punara	H	P	W	R	Gas troubles			Karak

*Camellia sinensis* (green tea) L. Kuntze. *Theaceae *	Sabz pati	H	P	C	L	Leaves are boiled in water mixed some sugar and take orally for indigestion and nausea	1 or half cup once a day	1 cup a day	D. I. Khan, Bannu, Lakki Marwat

*Carissa opaca *Stapf. ex Haines Apocynaceae	Granda	S	A	W/C	L, R, F	Dysentery			Karak

*Carum copticum* (L.) Benth. & Hook.f. ex C.B. Clarke Umbelliferae	Spaerkae	H	A	C	S	Used in sore throat, diarrhea, dysentery, and vomiting			Kohat

*Cassia fistula *L. Caesalpiniaceae	Amaltas	T	P	C	F	A piece of the fruit containing 10–12 seeds are slightly ground and boiled in 1/2 liter of water and strained for dysentery	1-2 tea spoons 3 times daily for 1-2 days	3-4 tea spoons 3-4 times daily	D. I. Khan, Bannu

*Cedrus deodara* (Roxb. ex D. Don) G. Don Pinaceae	Diar	T	A	C	Wh. pl	Gas troubles			Karak

*Cinnamomum zeylanicum* Blume Lauraceae	Dalchini	T	P	C	Ba	Bark powder and decoction is used in gastrointestinal disorder, vomiting, dysentery, and diarrhea	As needed	As needed	Kohat

*Citrullus colocynthis* (L.) Schrad. Cucurbitaceae	Kartumma	H	A	W	F	Fruit is cut, boiled in water and sugar added to make murabba used for constipation and abdominal diseases	As needed	As needed	D. I. Khan. Bannu, Kohat, Lakki Marwat

*Coriandrum sativum *L. Apiaceae	Dhania	H	A	C	F	Fruit is crushed and mixed with salt as carminative, indigestion	Small amount of safoof 2 times a day	Small amount of safoof 2 times a day	D. I. Khan

*Crotalaria burhia* Benth. Papilionaceae	Sassai	S	P	W	Wh. pl	Dried plant is ground, mixed with water, and strained and is given locally for diarrhea and other abdominal troubles			D. I. Khan

*Curcuma longa* L. Zingiberaceae	Haldi	H	A	C	Rh	Rhizome powder is effective in stomach cancer, stomach bleeding	Not used still	As needed	Kohat

*Cyperus rotundus *L. Cyperaceae	Delloca	H	P	W	Rh	Rhizome is effective in treatment of dyspepsia, diarrhea and vomiting	As needed	As needed	Bannu

*Datura stramonium* L. Solanaceae	Datura	H	A	W	Wh. pl	Seeds are used for intestinal worms	As needed	As needed	Karak

*Elaeagnus angustifolia* L. Cactaceae	Sersang	T	P	W	R, S, F	Constipation			Lakki Marwat

*Ephedra gerardiana *Wall ex Stapf. Ephedraceae	Somane	T	A	C/W	Sh, F, L	Dyspepsia			Lakki Marwat

*Equisetum arvense* L. Equisetaceae	Horstail, Chihly	H	P	W	Wh. pl	Diarrhea			Lakki Marwat

*Eugenia jambolana* Lam. Myrtaceae	Jaman	T	P	C	S	For stomach problems, grind the seeds and make powder	As needed	1-2 spoons daily for 3 days	Bannu

*Euphorbia hirta* L. Euphorbiaceae	Titra	H	P	W	Wh. pl	Stomach pain			Lakki Marwat

*Ficus religiosa* L. Moraceae	Peepal	T	P	W/C	Ba	Burn the bark and make powder from it. Take 5 grams of it orally with water for diarrhea	As needed	As needed	Bannu

*Foeniculum vulgare *Mill. Apiaceae	Saunf	H	A	C	F	Equal quantity of fennel fruit, coriander fruit, *Anethum sowa* and sugar are mixed and ground together to make powder for dyspepsia and abdominal pain	As required	Twice a day after meal	D. I. Khan

*Fumaria indica *(Hausskn.) Pugsley Fumariaceae	Shahtera	H	P	W	Wh. pl	It is used in aches and pains, diarrhea, vomiting	As needed	As needed	Kohat

*Lycium barbarum *L. Solanaceae		S	P	W	L	Extract from leaves is effective in curing bloody diarrhea and vomiting	As needed	As needed	Bannu

*Malva neglecta* Wallr. Malvaceae	Panerak	H	P	W	Sh	Stomach pain			Lakki Marwat

*Malva parviflora *L. Malvaceae	Puskay	H	P	W	L	Decoction of leaves is used for stomach problem. It is also used as laxative	Not used	As needed	Karak

*Melia azadirachta*L. Meliaceae	Dharek/Bakain	T	P	C/W	L, F	Leaves and fruit decoction is used to remove intestinal worms	Little amount is given, more dosage cause vomiting	As needed, more dosage cause vomiting	Lakki Marwat

*Mentha longifolia* (L.) L. Lamiaceae	Villanay, podina	H	A	C	Wh. pl	Fresh leaves are boiled in water with green tea mixed some sugar to cure diarrhea	2 tea spoons after 3 hours for 1 day	4 tea spoons after 3 hours for 1 day	Kohat, Lakki Marwat, Karak D. I. Khan

*Nasturtium officinale* R. Br. Brassicaceae	Termera	H	P	W	Wh. pl	Leaves are taken orally for constipation	As needed	As needed	D. I. Khan

*Opuntia dillenii *(Ker Gawl.) Haw. Cactaceae	Kunda thur	S	P	W	L, F	Ripened fruit is boiled in water add some sugar and take orally for constipation	As needed	As needed	D. I. Khan

*Opuntia monacantha *(Willd.) Haw. Cactaceae	Chnutarthar	S	P	W	Wh. pl	Digestion help			D. I. Khan

*Oxalis corniculata* L. Oxalidaceae	Tarookay	H	P	W	L	Extract juice from fresh leaves use orally against stomach troubles	As needed	As needed	Bannu

*Phyllanthus emblica* Phyllanthaceae	Amla	T	P	W	F	Effective in diarrhea dysentery			Kohat

*Punica granatum* L. Punicaceae	Anar	T	P	C	F	Outer covering of fruit is dried and crushed and powder is taken with water for diarrhea	One table spoon daily for 2-3 days	One table spoon daily for 2-3 days	D. I. Khan

*Rosa indica* L. Rosaceae	Gulab	S	P	C	Fl	Flower are mixed with sugar put in sun place take orally with water and fennel for vomiting and dyspepsia	2 g twice a day	5 g twice a day	D. I. Khan, Bannu, Kohat

*Saccharum officinarum* L. Poaceae	Gana	S	A	C	St.	Stem extract is useful in indigestion	Twice a day	Twice a day	D. I. Khan, Lakki Marwat Bannu

*Sisymbrium irio *Linn. Brassicaceae	Shonopy	H	A	W	L	Leaves are used for stomach problems	As needed	As needed	Karak

*Solanumsurattense *Burm. F Solanaceae	Manraghonay	H	B	W	F	Fruit is dried, crushed and powder is taken for abdomen pain and gas trouble	As needed	As needed	Bannu, Kohat

*Tordylium nodosum* L. Apiaceae	Hoso beta	H	A	W	Wh. pl	Intestinal worms			Bannu

*Withania coagulans* (Stocks) Dunal Solanaceae	Khapyanga, Paneer	H	A	W	F, L	Fruits are crushed, mix with salt and take orally with water for gastric and abdominal pain. Fruits are stained in water and taken. Extract of leaves is used	Twice a day for 2-3 days	Thrice a day for 3-4 days	Karak, Kohat, D. I. Khan, Lakki Marwat, Bannu

*Woodfordia fruticosa *(L.) Kurz Lythraceae	Dhawai	S	P	W/C	Fl	It is used in diarrhea, dysentery, ulcers, and UTI			Kohat

*Ziziphus jujuba* Mill. Rhamnaceae	Jangli bera	T	P	W/C	F	Roast the fruit and eat for the treatment of stomach problems. Take 5 grams of root powder and 7 pieces of black pepper, grind and mix used to cure diarrhea and abdominal pain	Small amount twice a day	400 mg thrice a day for 3 days	Bannu, D. I. Khan, Lakki Marwat

*Ziziphus nummularia* (Burm. f.) Wight & Arn. Rhamnaceae	Bair	T	P	C	F	Decoction of leaves and bark is used in dysentery	As needed	Take orally thrice a day for 3-4 days	Bannu, D. I. Khan, Kohat

T: tree, S: shrub, H: herb, P: perennial, A: annual, B: biennial, W: wild, C: cultivated, F: fruit, L: leaves, Fl: flower, Wh. Pl: whole plant, St: stem, Sh: shoot, Ba: bark, S: seed, R: root, Rh: rhizome, Po: pods, and Bu: bulb.

**Table 2:** Habit, life-span, and parts used of medicinal plants.

General attributes	Total plants	% age
Habit		
Herb	26	50
Tree	16	31
Shrub	10	19
Life-span		
Perennial	32	62
Annual	17	33
Biennial	3	6
Part Used		
Fruit	15	24
Whole plant	14	23
Leaves	12	19
Bark	5	8
Root	4	6
Seeds	3	5
Flower, shoot, and rhizome	2	3
Pods, stem, and bulb	1	2
Growth form		
Wild	35	60
Cultivated	23	40

**Table 3:** FIC values of traditional medicinal plants for treating stomach problems in study regions.

S. number	Disease category	Plant species	Number of taxa (Nt)	Number of use reports (Nur)	FIC
1	Vomiting and nausea	*Artemisia absinthium* (5), *Camellia sinensis* (20), *Carum copticum* (10), * Cinnamomum zeylanicum* (5), *Cyperus rotundus* (4), *Foeniculum vulgare* (25), *Lycium barbarum* (8), and *Rosa indica* (12)	8	89	0.92

2	Abdominal pain	*Achyranthes aspera* (3), *Artemisia absinthium* (8), *Asparagus adscendens *(6), * Citrullus colocynthis* (16), *Crotalaria burhia* (3), *Foeniculum vulgare* (10), *Solanum surattense* (10), *Camellia sinensis* (20), *Coriandrum sativum* (5), *Opuntia monacantha* (3), *Withania coagulans* (31), *Ziziphus jujuba* (13), *Azadirachta indica* (13), *Boerhaavia diffusa* (5), *Cinnamomum zeylanicum* (4), *Cyperus rotundus* (5), *Ephedra gerardiana*(4), *Foeniculum vulgare* (8), *Rosa indica* (15), and *Saccharum officinarum* (9)	20	191	0.9

3	Diarrhea	*Acacia nilotica* (5), *Albizia lebbeck* (8), *Allium cepa* (10), *Argyrolobium roseum* (6), *Artemisia absinthium* (7), *Carum copticum* (9), *Cinnamomum zeylanicum* (3), *Crotalaria burhia* (8), *Cyperus rotundus* (6), *Equisetum arvense* (5), *Ficus religiosa* (8), *Foeniculum vulgare* (10), *Lycium barbarum* (5), *Mentha longifolia* (30), *Ziziphus jujube* (18), *Phyllanthus emblica* (5), *Punica granatum* (8), and *Woodfordia fruticosa* (5)	18	156	0.89

4	Dysentery	*Achyranthes aspera* (5), *Carissa opaca* (8), *Carum copticum * (8), *Cassia fistula* (22), *Cinnamomum zeylanicum* (3), *Phyllanthus emblica* (5), *Woodfordia fruticosa* (5), *Ziziphus nummularia* (13)	8	69	0.89

5	Intestinal worms	*Artemisia absinthium* (6), *Datura stramonium* (7), *Melia azadirachta* (9), and *Tordylium nodosum* (3)	4	25	0.87

6	Constipation	*Abutilon indicum* (3), *Acacia nilotica* (5), *Bistorta amplexicaulis* (5), *Citrullus colocynthis* (16), *Elaeagnus angustifolia* (4), *Nasturtium officinale* (7), and *Opuntia dillenii* (5)	7	45	0.86

**Table 4:** Fidelity level (FL) values for common medicinal plants used by local traditional healers by ailment category.

S. number	Plant spp.	Disease category	Ip	Iu	FL%
1	*Rosa indica *	Vomiting and nausea	12	30	66
2	*Withania coagulans *	Abdominal pain	31	36	86
3	*Mentha longifolia *	Diarrhea	30	40	75
4	*Foeniculum vulgare *	Diarrhea	10	17	58
5	*Phyllanthus emblica *	Dysentery	5	13	38
6	*Melia azadirachta *	Intestinal worms	9	12	75
7	*Citrullus colocynthis *	Constipation	16	22	72

**Table 5:** Demographic profile of the informants.

Demographic characters	Total	Percentage
Gender		
Male	200	57
Female	150	43
Age groups		
21–29	30	9
30–39	60	17
40–49	100	29
50–59	90	25
60–69	40	11
70–79	30	9
Education		
Illiterate	155	44
Primary	85	24
Middle	50	14
Secondary	45	13
University	15	4
*Occupation *		
Female		
House wives	80	23
Primary teachers	50	14
Secondary teachers	20	6
Males		
Farmers	90	26
Shopkeepers	40	11
Labourers	50	14
School teachers	20	6
